# Topical Minoxidil and Low‐Dose Oral Minoxidil in Onychodystrophy: A Proposed Treatment Algorithm

**DOI:** 10.1111/jocd.70863

**Published:** 2026-04-19

**Authors:** Aditya K. Gupta, Mesbah Talukder, Shari R. Lipner

**Affiliations:** ^1^ Mediprobe Research Inc. London Ontario Canada; ^2^ Division of Dermatology, Temerty Faculty of Medicine University of Toronto Toronto Canada; ^3^ School of Pharmacy BRAC University Dhaka Bangladesh; ^4^ Department of Dermatology, Weill Cornell Medicine New York City New York USA

**Keywords:** minoxidil, nail growth, onychodystrophy, onychomadesis and nail psoriasis, yellow nail syndrome

## Abstract

**Background:**

Onychodystrophy includes diverse nail disorders that may impair function and quality of life. Because nails grow slowly, adjunctive therapies that promote nail growth are of increasing interest.

**Aim:**

This review examines the evidence for minoxidil in nail growth and nail disorders and proposes a treatment algorithm for its adjunctive use in onychodystrophy.

**Method:**

A structured search of PubMed and Google Scholar was conducted on December 10, 2025, using terms related to minoxidil and nails. Records were deduplicated, screened, and appraised by full‐text review using the Oxford Centre for Evidence‐Based Medicine 2009 levels.

**Results:**

A potential adjunctive treatment algorithm is proposed, emphasizing treatment of the underlying nail disorder first. Topical minoxidil (2% or 5% solution or foam, applied once or twice daily to the proximal nail fold) or low‐dose oral minoxidil (LDOM; 0.625–2.5 mg/day in females and 1.25–5 mg/day in males) may then be considered, with appropriate monitoring and discontinuation criteria. Safety data for topical use indicate predominantly local irritation, whereas LDOM has been associated with tachycardia, edema, and other systemic adverse effects. Minoxidil may support nail growth through KATP channel‐mediated vasodilation and growth factor signaling, with downstream activation of the Wnt/β‐catenin pathway.

**Conclusion:**

Minoxidil shows potential as an adjunct to support nail growth, but nail‐disorder–specific evidence remains limited. Controlled trials are needed to define efficacy, optimal regimens, and safety in onychodystrophy.

## 1 Introduction

Onychodystrophy refers to a diverse group of nail disorders marked by abnormalities in one or more of the following: Nail plate morphology, color, thickness, surface texture, or growth [1, 2]. These changes may arise from nail matrix dysfunction due to inflammatory dermatoses, infection, trauma, medication effects, or systemic disease [1, 2]. Nail disorders can cause pain, impair function, and substantially affect quality of life. In addition, nails, particularly toenails, grow slowly. Therefore, clinical improvement is often delayed even after the underlying cause is treated, and recovery may require many months for the dystrophic nail plate to be partially or fully replaced [3].

Minoxidil, a potent vasodilator, is established as a topical therapy for androgenetic alopecia and is increasingly used off‐label at low oral doses (0.625 mg to 5 mg/day) for hair loss disorders [4, 5, 6, 7, 8]. Beyond hair disorders, case reports, personal experience, and controlled studies in healthy volunteers indicate that topical minoxidil application to the nail matrix area can increase fingernail growth rates over weeks [9, 10, 11, 12]. Early clinical reports further suggest the potential utility of minoxidil application in slow‐growth nail disorders such as the yellow nail syndrome (YNS) and nail growth arrest, though evidence is largely observational and often confounded by concomitant therapies [12, 13, 14]. This review summarizes available clinical evidence, outlines plausible mechanisms, and discusses practical considerations for off‐label use of minoxidil and safety in patients with onychodystrophy.

## 2 Search Strategy and Methodology

**FIGURE 1:**
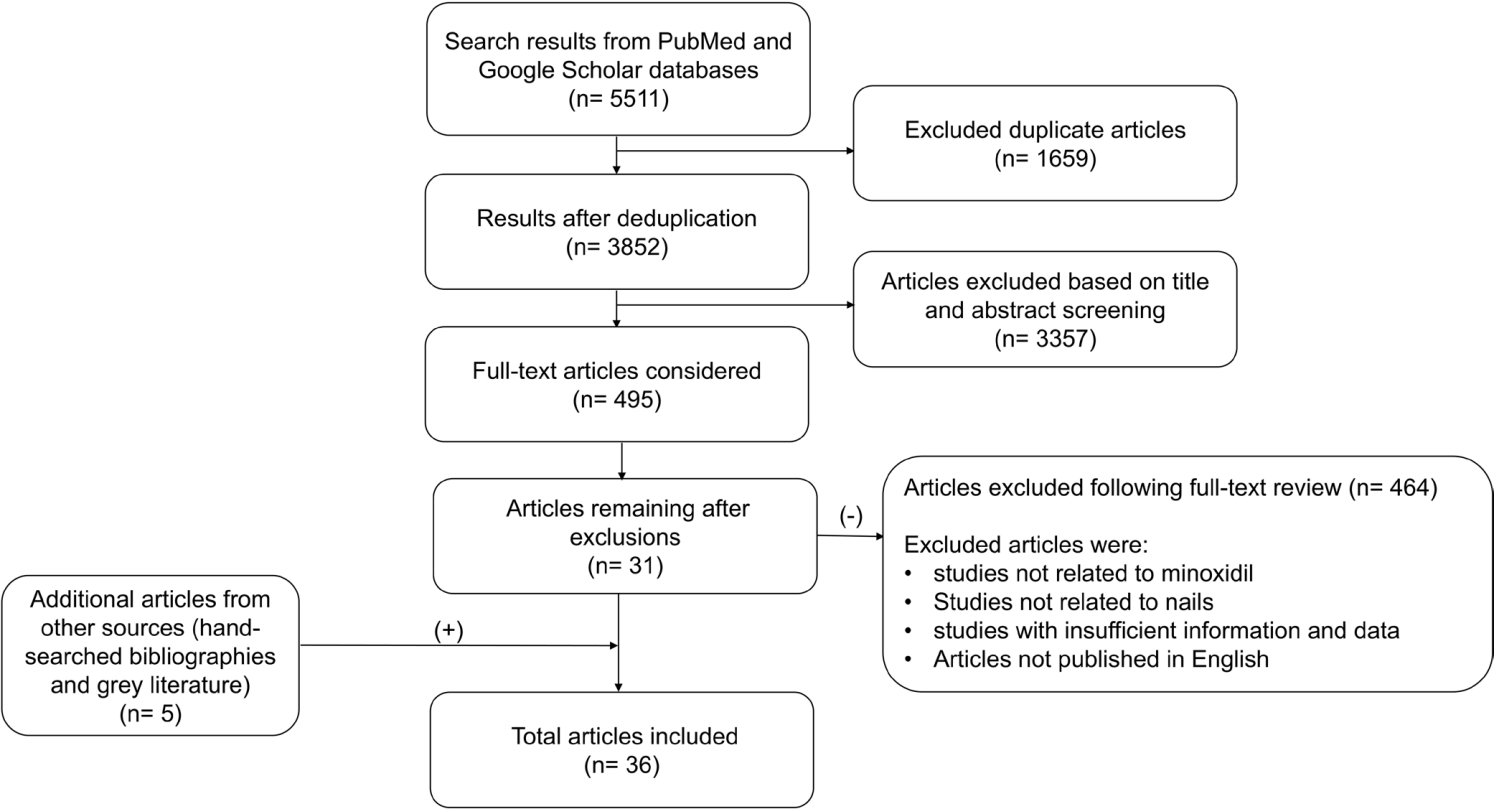
Study selection flow diagram.

A structured literature search was conducted on December 10, 2025, using PubMed and Google Scholar. The following keywords were used to conduct the search: Minoxidil, nail growth, onychodystrophy, yellow nail syndrome, onychomadesis, and nail psoriasis. Records from both databases were imported and deduplicated. Titles and abstracts were screened for relevance to minoxidil and nail disorders or nail outcomes. Full texts were assessed for eligibility. Figure 1 presents the exclusion criteria of the articles. The quality of the evidence was evaluated according to the 2009 Oxford Centre for Evidence‐Based Medicine (OCEBM) standards [15].

## 3 Clinical Evidence

Clinical studies on the effects of minoxidil on nails can be broadly divided into two groups: (a) controlled studies in healthy volunteers showing that minoxidil can accelerate nail growth, and (b) clinical reports involving nail disorders, such as YNS (Table 1) [9, 10, 11, 12, 13, 14, 16, 17]. A 2025 systematic review synthesized six minoxidil‐associated studies (*n* = 197) and concluded that early data support possible improvements in nail outcomes but emphasized heterogeneity and limited high‐quality disease‐focused trials [18].

**TABLE 1:** Summary of clinical evidence evaluating minoxidil for nail disorders and nail‐growth outcomes.

Author (year)	Study type	Evidence level	Sample size	Type of nail disorder	Treatment regimen	Outcome measures	Outcome/results	Side effect	Comment
Desai (2025) [14]	Case series (2 cases)	4	2 (1 M, 1F)	Yellow nail syndrome (YNS)	Oral minoxidil; Case 1 (male): 0.625 mg BID; Case 2 (female): 1.25 mg daily	Clinical appearance (yellowing, onycholysis, onychomadesis), visible proximal regrowth; patient report of trimming frequency	Visible improvement by ~3 months; case 1 continued improvement to 6 months with new proximal plate; case 2 improved by 3 months with ongoing improvement afterward	None reported	Promising signal for YNS nail changes with low‐dose oral minoxidil (LDOM); evidence is limited to case observations
Barbosa (2024) [16]	Open study	2b	10 M	Healthy nails (growth model)	Oral minoxidil 1 mg and 2.5 mg daily for 14 days	Change in nail growth speed via dermoscopic photos/ImageJ; vitals monitoring	2.5 mg increased nail growth speed by 50.7% (*p* < 0.01); 1 mg no significant increase	HR increased (dose‐related), no BP change	Provides controlled measurement evidence that higher‐dose LDOM can accelerate nail growth in men
Starace (2023) [12]	Retrospective study	4	50 (4 M, 46F)	Toenail growth arrest/onychomadesis	Daily 5% topical minoxidil “drop” on proximal nail fold of involved toenails + vitamin E (3 × 400 IU daily) for 12 months	Clinical exam/photo + standardized nail growth; response score at 6 and 12 months	At 6 months: No response (7%), **mild response (59%)**, good response (26%), optimal response (8%) At 12 months: No response (1%), mild response (18%), **good response (46%)**, optimal response (35%)	None reported	Suggests meaningful clinical improvement by 12 months, but confounded by concomitant vitamin E, retrospective design, and subjective response scoring.
Nitayavardhana (2023) [10]	Randomized controlled trial	1b	30 (4 M, 26F)	Healthy nails (growth model)	Randomized to 2% minoxidil vs. 5% minoxidil vs. placebo, applied BID to proximal nail folds of 2nd and 4th fingers; assessed at week 4 and 8	Nail growth rate at weeks 4 and 8; adverse reactions	At week 8: 2% had higher growth (3.32 ± 0.63 mm) vs. 5% (2.88 ± 0.50 mm) and placebo (2.87 ± 0.54 mm)	No cutaneous/systemic side effects observed	Suggests concentration‐response is not linear for nail growth (2% > 5% here); healthy volunteers, so translation to disease is uncertain.
Alsalhi (2023) [17]	Cross‐sectional	4	66 (71.2% female)	Mostly normal nails (2 had prior toenail onychomycosis)	Oral minoxidil 0.25–5 mg/day (median dose 1.25 mg, median duration ~10.7 months at survey time)	Self‐reported changes in growth, strength, appearance	53% reported faster growth; 37.9% stronger nails; 36.4% nicer appearance	Not reported	Suggests a patient‐perceived nail benefit with oral minoxidil, but subjective and uncontrolled.
Garbers (2021) [11]	Quasi‐experimental open controlled factorial study (healthy adults)	2b	38 (Both M and F, but the numbers were not specified.)	Healthy nails	14‐day baseline, then groups including 5% topical minoxidil spray BID, biotin 2.5 mg/day, combination, or control; follow‐up to day 28	Nail growth rate (mm/day) via dermoscopy/photos	Nail growth higher in intervention groups; at day 28, minoxidil group showed ~19% increase vs. control, outperforming biotin (13%) and the combination (14%)	None reported	Supports growth acceleration with topical minoxidil in healthy adults; short duration and healthy sample limit generalizability to nail disorder.
Algain (2021) [13]	Case report	4	1F	Yellow nail syndrome (discoloration)	Topical minoxidil 2% plus terbinafine 250 mg/day	Clinical nail appearance and growth; degree of improvement; persistence of discoloration; recurrence over follow‐up	After adding topical minoxidil and continued terbinafine, at ~6 months: Yellow persisted only on distal half of both big toes; later complete remission in all nails except big toes; no recurrence over 72 months	None reported	Strong clinical response, but highly confounded (multiple systemic agents; minoxidil added only after partial response to terbinafine).
Aiempanakit (2017) [9]	Pilot experimental study	2b	32 (16 M, 16F)	Healthy nails	5% topical minoxidil to selected fingernails BID for 8 weeks; treated nails vs. untreated nails	Caliper‐measured nail length change from baseline; vitals & cutaneous adverse effects	Treated nails grew faster than untreated; e.g., week 4 4.27 vs. 3.91 mm (*p* = 0.001) and week 8 7.46 vs. 6.93 mm (*p* < 0.001)	None reported	Supports biologic plausibility for nail slow growth disorders; indirect evidence (healthy nails) and no placebo control

Abbreviations: M: Male, F: Female, BID: Twice daily, 1b = individual RCT, 2b = individual cohort or low‐quality RCT, 2c = outcomes research, 4 = case series/case report.

### 3.1 Topical Minoxidil on Healthy Nails

Aiempanakit et al. conducted a pilot study evaluating whether topical 5% minoxidil accelerates fingernail growth [9] (Table 1). In the study, 5% topical minoxidil was applied twice daily to fingernails in 32 healthy participants (16 male and 16 female), and linear growth of the nails was measured over time. It was observed that treated nails grew faster than untreated nails, with differences apparent early and sustained through follow‐up [9]. The investigators reported no systemic or cutaneous side effects in participants and did not observe meaningful vital sign changes, supporting a favorable short‐term safety profile for localized proximal nail‐fold application [9]. Although this study provides biologic plausibility for growth acceleration, it does not establish clinical efficacy for onychodystrophy since participants did not have nail disease.

Nitayavardhana and colleagues performed a randomized study comparing topical 2% minoxidil, topical 5% minoxidil, and placebo for the fingernail growth rate [10] (Table 1). Thirty participants (4 males and 26 females) were randomized into three groups and instructed to apply the solution twice daily to specified fingers; nail lengths were measured at weeks 4 and 8 using digital calipers, and possible side effects were assessed by a dermatologist [10]. The study reported that at week 8, the 2% minoxidil group demonstrated a higher mean nail growth rate than both the 5% minoxidil and placebo, and no adverse reactions were observed [10]. This trial provides the strongest controlled evidence that topical minoxidil can increase nail growth rate under standardized conditions.

Garbers and colleagues evaluated nail growth rate in 38 healthy adults using an open, controlled factorial design over 28 days, comparing topical 5% minoxidil, oral biotin (2.5 mg), combination therapy, and controlled conditions [11] (Table 1). Nail growth was assessed using dermoscopy/photographic methods and ImageJ‐based measurement [11]. The authors reported an increase in nail growth rate with topical minoxidil compared with control and noted no dropouts or reported adverse events over the brief follow‐up [11]. While limited by short duration and a non‐disease population, this study further supports growth acceleration and provides a comparative context against a commonly used supplement.

### 3.2 Oral Minoxidil on Healthy Nails

Alsalhi et al. conducted a cross‐sectional analysis of 66 patients taking oral minoxidil for hair loss and assessed self‐reported nail changes, including perceived growth and strength [17] (Table 1). Approximately half of the respondents reported faster nail growth, and a substantial minority reported stronger or improved‐appearing nails while using oral minoxidil [17]. This study suggests a perceived nail effect in oral minoxidil users.

### 3.3 Topical Minoxidil for YNS

Algain reported a case (female patient) of YNS that eventually improved following a regimen that included systemic antifungals and adjunctive topical minoxidil 2% applied around selected non‐responding nails [13] (Table 1). Long‐term follow‐up noted sustained improvement with minimal adverse effects under monitoring [13]. The regimen involved multiple concomitant therapies; therefore, it is hard to identify the benefit of minoxidil on its own [13].

### 3.4 Topical Minoxidil for Onychomadesis

Starace et al. described a retrospective study of 50 patients with nail growth disorders, including growth arrest and onychomadesis, using topical 5% minoxidil applied to the proximal nail fold alongside vitamin E over a prolonged interval [12] (Table 1). The authors reported progressive improvement over time based on their scoring approach [12]. Interpretation is limited by retrospective design, lack of a control group, and concomitant vitamin E therapy. However, the study aligns with the concept that sustained application of topical minoxidil may be required for clinically meaningful toenail outcomes.

### 3.5 Oral Minoxidil for YNS

Desai et al. reported two cases of YNS with improvement in nail changes using low‐dose oral minoxidil over 3–6 months, with therapy reportedly well tolerated and visible nail improvement (Table 1) [14]. These cases are notable because they propose oral minoxidil as a potentially novel approach for YNS nail manifestations.

## 4 Discussion

### 4.1 Clinical Relevance of Minoxidil for Onychodystrophy

The most consistent signal across studies is that minoxidil can accelerate nail growth in healthy volunteers under controlled conditions [9, 10, 11, 12]. This supports biologic plausibility and provides a rationale for exploring clinical benefit in slow‐growth nail disorders. However, the evidence for true onychodystrophy treatment remains limited. Most disease‐focused reports are uncontrolled and frequently confounded by concomitant therapies [12, 13, 14]. As such, minoxidil should not be considered as a general treatment for onychomycosis‐like dystrophy or inflammatory nail dystrophy unless disease‐specific trials demonstrate benefit. Instead, minoxidil can be used as an adjunct for slow‐growth phenotypes (growth arrest, onychomadesis, YNS).

### 4.2 Proposed Treatment Algorithm

**FIGURE 2:**
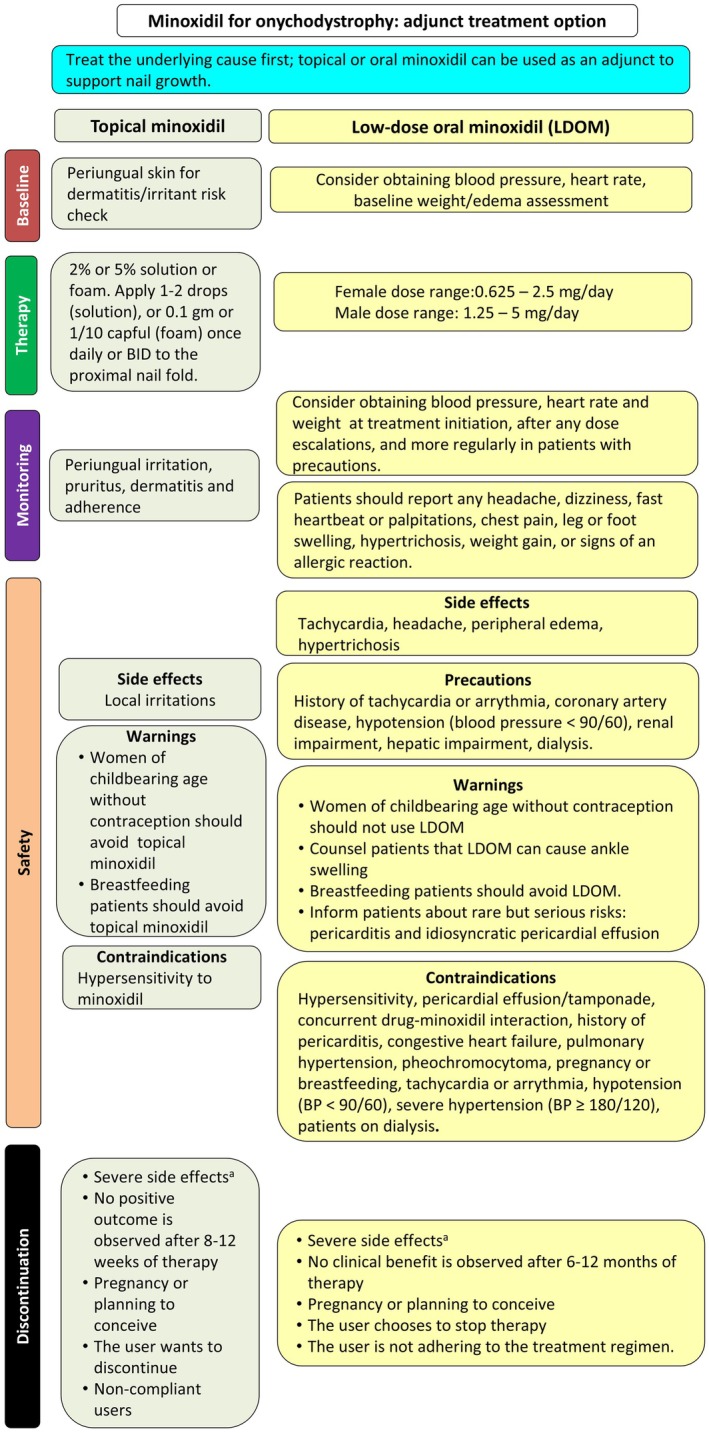
Proposed treatment algorithm for onychodystrophy using topical minoxidil or low‐dose oral minoxidil (LDOM) as an adjunctive treatment option. ^a^Chest pain, faintness or dizziness, unexplained weight gain, swollen hands or feet, persistent local redness or rash, shortness of breath.

First, the underlying cause of onychodystrophy (e.g., inflammatory disease, infection, or trauma) should be treated. Topical or oral minoxidil may be considered as a nail‐growth supporting option (Figure 2). For topical minoxidil, screen periungual skin for irritant or dermatitis risk; apply 2% or 5% solution (1–2 drops) or foam (0.1 g≈1/10 capful) to the proximal nail fold once daily or twice daily; and monitor for local irritation, pruritus, dermatitis, and adherence [9, 10, 11, 12, 19]. For low‐dose oral minoxidil (LDOM), physicians should consider obtaining baseline blood pressure, heart rate, and weight/edema assessment; regimens include minoxidil 0.625 mg twice daily or 1.25 mg daily [14]. Doses can be titrated upwards with possible ranges in females being 0.625–2.5 mg/day and 1.25–5 mg/day in males [4, 5]. Physicians should counsel patients to report headache, dizziness, palpitations, chest pain, peripheral edema, hypertrichosis, weight gain, or allergic reactions [14]. The precautions of LDOM include arrhythmia, coronary disease, hypotension, renal/hepatic impairment, or dialysis. LDOM should be avoided in pregnancy or breastfeeding; rare serious risks include pericarditis and pericardial effusion [20, 21, 22]. Minoxidil should be discontinued in the event of severe adverse effects, such as faintness or dizziness, unexplained weight gain, swelling of the hands or feet, persistent local redness or rash, or shortness of breath, chest pain, or in cases of lack of benefit, pregnancy, or non‐compliance [20, 21, 22].

### 4.3 Topical Minoxidil Delivery Considerations

Topical therapy for nail targets is constrained by the nail unit barrier. The nail plate is a dense keratin barrier, and successful topical delivery often requires targeted application sites, optimized formulations, and sometimes permeation strategies [23, 24]. In nail growth trials, minoxidil is typically applied to the proximal nail fold rather than the nail plate itself, likely aiming to influence the matrix region through periungual skin rather than transungual diffusion [9, 10, 12]. For clinical use, periungual application to the proximal fold is more consistent with the existing evidence base than applying minoxidil onto the nail plate surface.

### 4.4 Limitations

The number of studies evaluating minoxidil for the treatment of onychodystrophy is limited. Moreover, many clinical reports involve concomitant therapies, making it difficult to isolate the effect of minoxidil alone [12, 13]. Safety in nail‐disorder populations, particularly with oral use, is largely extrapolated from alopecia cohorts and product labeling rather than dedicated nail trials. Additionally, publication bias and selective reporting may inflate the perceived benefit.

## 5 Mechanisms

### 5.1 Minoxidil Is a Vasodilator

**FIGURE 3:**
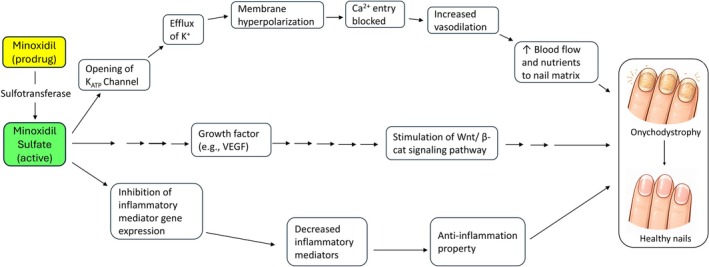
Proposed mechanisms by which minoxidil may support nail growth and improve onychodystrophy. Minoxidil (prodrug) is converted by sulfotransferase to active minoxidil sulfate, which opens ATP‐sensitive potassium (K_ATP_) channels, leading to K^+^ efflux, membrane hyperpolarization, reduced Ca^2+^ entry, and vasodilation. Vasodilation may increase blood flow and nutrient delivery to the nail matrix, supporting nail growth. In addition, minoxidil may upregulate growth factors (e.g., VEGF) and stimulate Wnt/β‐catenin signaling, potentially promoting matrix activity. Minoxidil may also decrease inflammatory mediators by reducing inflammatory mediator gene expression and provide anti‐inflammatory effects that could further support nail growth. VEGF, Vascular Endothelial Growth Factor. Wnt/β‐catenin, Wingless/Integrated (Wnt) signaling pathway with β‐catenin.

Minoxidil may accelerate nail growth through vasodilation and increased blood flow to the nail unit [13, 14, 25] (Figure 3). It is a prodrug that requires bioactivation (sulfation) to minoxidil sulfate, its active metabolite. Minoxidil sulfate stimulates the opening of ATP‐sensitive potassium (K_ATP_) channels [26]. The K_ATP_ channel opening promotes K^+^ efflux and membrane hyperpolarization, which reduces intracellular Ca^2+^ (via reduced Ca^2+^ influx through voltage‐dependent channels) and results in vasodilation [26].

### 5.2 Minoxidil Upregulates Growth Factors

A key mechanistic thread is minoxidil's capacity to upregulate VEGF (vascular endothelial growth factor) in dermal papilla cells, supporting angiogenesis and potentially increased nutritive blood flow [27] (Figure 3).

### 5.3 Anti‐Inflammatory Property of Minoxidil

There is evidence that controlling inflammation can improve nail growth, particularly in inflammatory nail disorders such as nail psoriasis and chronic paronychia [28, 29]. Therefore, the anti‐inflammatory effects could plausibly support nail matrix function and enhance nail growth (Figure 3).

## 6 Safety

### 6.1 Oral Minoxidil Safety

At standard antihypertensive doses, typically 10–40 mg/day, oral minoxidil can present significant systemic risks, notably reflex tachycardia and fluid retention. Consequently, it requires close clinical supervision and the use of co‐therapies, such as diuretics and beta‐blockers, to manage these side effects in hypertension management [21].

In dermatology and hair restoration, low‐dose oral minoxidil (LDOM) (0.625–5 mg/day) is widely used off‐label for hair loss. A multicenter study of 1 404 patients found that systemic adverse effects were infrequent, with a low discontinuation rate due to adverse events, supporting a generally favorable safety profile in appropriately selected patients [22]. Nevertheless, nail‐disorder use is typically off‐label and evidence‐limited; therefore, risk–benefit discussions should be conservative. Physicians should counsel about potential adverse effects (e.g., tachycardia/palpitations, headache, dizziness, peripheral edema, and hypertrichosis) and consider baseline blood pressure/heart rate assessment with follow‐up monitoring, especially in patients with cardiovascular or renal comorbidities [22].

### 6.2 Topical Minoxidil Safety

Topical minoxidil labeling includes warnings about systemic‐type symptoms, e.g., low blood pressure, rapid heartbeat, dizziness, extremity swelling, chest pain, weight gain, with medical evaluation and discontinuation if these occur, reflecting the possibility of systemic absorption and sensitivity in some users [30]. Nonetheless, systemic absorption from topical minoxidil applied to skin is generally low, and nail‐growth studies applying minoxidil to the proximal nail fold reported no meaningful systemic effects in controlled settings [9, 10, 11]. Local irritation or contact dermatitis remains a plausible risk, especially with alcohol/propylene glycol vehicles [30]. In nail applications, careful counseling should include periungual irritation monitoring and avoidance of application on broken/inflamed periungual skin.

### 6.3 Special Populations

#### 6.3.1 Renal Impairment

The oral minoxidil product monograph notes that patients with renal failure or those on dialysis may require dose adjustment and careful supervision to prevent exacerbation of renal failure or precipitation of cardiac failure [20]. For nail indications, where benefit is uncertain, systemic therapy should be approached cautiously in chronic kidney disease, with preference for topical therapy when feasible.

#### 6.3.2 Hepatic Impairment

Oral minoxidil in patients with severe hepatic impairment is contraindicated [20]. Topical minoxidil therapy would be preferred in this setting; however, if oral minoxidil is considered for nail use in patients with mild‐to‐moderate hepatic impairment, initiating at the lowest dose with close monitoring is prudent [20].

#### 6.3.3 Cardiac Impairment

Oral minoxidil's hemodynamic effects (e.g., blood pressure and heart rate) are clinically relevant in heart failure, coronary disease, arrhythmia susceptibility, or pericardial disease; labeling underscores careful supervision in systemic use [6, 20, 31]. For off‐label nail indications, oral minoxidil should generally be avoided or used only with specialist involvement in patients with significant cardiac disease, especially when safer alternatives exist.

#### 6.3.4 Pregnancy

The package insert of women's topical minoxidil indicates that it should not be used during pregnancy [32]. Oral minoxidil labeling indicates that safe use in pregnancy is not established (pregnancy category C) and recommends use only if the potential benefit justifies fetal risk [21]. For nail indications, the avoidance of the use of minoxidil during pregnancy is recommended.

#### 6.3.5 Breastfeeding

Topical minoxidil used by the lactating mother likely poses low risk to older, full‐term infants but should be used cautiously, and avoidance may be preferable when breastfeeding a preterm or neonatal infant [33]. The database also summarizes case reports suggesting possible infant effects (e.g., hypertrichosis) in some circumstances [33]. Oral minoxidil labeling advises against use in nursing women due to potential adverse effects in infants and reports excretion into breast milk [21].

#### 6.3.6 Pediatric Patients

Systemic minoxidil experience in pediatric populations is limited, and labeling recommends careful titration [21]. Given the limited nail‐specific evidence and availability of alternative nail care strategies, pediatric nail use of minoxidil (especially oral) should be used with caution and supervised by a pediatrician.

#### 6.3.7 Geriatric Patients

Product monograph labeling notes limited trial representation and recommends cautious dosing due to comorbidity burden [21]. This is particularly relevant because YNS often occurs in older adults and may coexist with respiratory or lymphatic disease [34], complicating oral minoxidil risk assessment.

## 7 Conclusion

Minoxidil has emerging evidence as a nail growth modulator. Controlled studies demonstrate that topical minoxidil applied to the proximal nail fold can increase linear nail growth within weeks. Additionally, low‐level clinical evidence suggests a possible benefit in slow‐growth nail disorders such as YNS. Given limited disease‐specific evidence and the systemic risks of oral minoxidil, topical therapy should be prioritized when considering off‐label use, and oral therapy should be reserved for carefully selected patients with monitoring and shared decision‐making guided by labeling and broader dermatology safety data. Future standardized, disease‐focused randomized trials are required to define efficacy, optimal concentration, duration, and safety in onychodystrophy.

## Author Contributions

The conception of the manuscript was done by Aditya K. Gupta (AKG). The manuscript was drafted by Aditya K. Gupta (AKG), Mesbah Talukder (MT), and Shari R. Lipner (SRL). The manuscript was substantively edited and revised by Aditya K. Gupta (AKG), Mesbah Talukder (MT), and Shari R. Lipner (SRL).

## Funding

The authors have nothing to report.

## Disclosure

Figure 3 provides general information only and does not substitute for professional medical judgment. Clinical decisions should be based on the most recent therapeutic guidelines, official prescribing information, and peer‐reviewed literature.

## Ethics Statement

Approval from an ethics board was not required as there was no direct involvement with human participants.

## Conflicts of Interest

The authors declare no conflicts of interest.

## Data Availability

Data sharing not applicable to this article as no datasets were generated or analysed during the current study.
